# Intimate Partners Violence against Women during a COVID-19 Lockdown Period: Results of an Online Survey in 7 Provinces of the Democratic Republic of Congo

**DOI:** 10.3390/ijerph18105108

**Published:** 2021-05-12

**Authors:** John D. Ditekemena, Christophe Luhata, Hypolite M. Mavoko, Joseph Nelson Siewe Fodjo, Dalau M. Nkamba, Wim Van Damme, Shahul H. Ebrahim, Christiana Noestlinger, Robert Colebunders

**Affiliations:** 1 Kinshasa School of Public Health, Faculty of Medicine, University of Kinshasa, Kinshasa 834, Congo; christophe.luhata@pevrdcongo.cd (C.L.); dalau.nkamba@unikin.ac.cd (D.M.N.); 2 Department of Tropical Medicine, Faculty of Medicine, University of Kinshasa, Kinshasa 834, Congo; hypolite.muhindo@unikin.ac.cd; 3 Global Health Institute, University of Antwerp, 2000 Antwerp, Belgium; JosephNelson.SieweFodjo@uantwerpen.be (J.N.S.F.); robert.colebunders@uantwerpen.be (R.C.); 4 Pôle d’Épidémiologie et Biostatistique, Institut de Recherche Expérimentale et Clinique (IREC), Université catholique de Louvain (UCLouvain), 1348 Brussels, Belgium; 5 Department of Public Health, Institute of Tropical Medicine, 2000 Antwerp, Belgium; wvdamme@itg.be (W.V.D.); cnoestlinger@itg.be (C.N.); 6 Technique and Technology, University of Sciences, Bamako 1805, Mali; ebrahimsh2@gmail.com

**Keywords:** COVID-19, lockdown, Intimate Partners’ Violence (IPV), DRC

## Abstract

Intimate Partners’ Violence (IPV) is a public health problem with long-lasting mental and physical health consequences for victims and their families. As evidence has been increasing that COVID-19 lockdown measures may exacerbate IPV, our study sought to describe the magnitude of IPV in women and identify associated determinants. An online survey was conducted in the Democratic Republic of Congo (DRC) from 24 August to 8 September 2020. Of the 4160 respondents, 2002 eligible women were included in the data analysis. Their mean age was 36.3 (SD: 8.2). Most women (65.8%) were younger than 40 years old. Prevalence of any form of IPV was 11.7%. Being in the 30–39 and >50 years’ age groups (OR = 0.66, CI: 0.46–0.95; *p* = 0.026 and OR = 0.23, CI: 0.11–048; *p* < 0.001, respectively), living in urban setting (OR = 0.63, CI: 0.41–0.99; *p* = 0.047), and belonging to the middle socioeconomic class (OR = 0.48, CI: 0.29–0.79; *p* = 0.003) significantly decreased the odds for experiencing IPV. Lower socioeconomic status (OR = 1.84, CI: 1.04–3.24; *p* = 0.035) and being pregnant (OR = 1.63, CI: 1.16–2.29; *p* = 0.005) or uncertain of pregnancy status (OR = 2.01, CI: 1.17–3.44; *p* = 0.011) significantly increased the odds for reporting IPV. Additional qualitative research is needed to identify the underlying reasons and mechanisms of IPV in order to develop and implement prevention interventions.

## 1. Introduction

Since the SARS CoV-2 outbreak in December 2019, countries around the world have put in place outbreak-control measures [1]. The government of the Democratic Republic of Congo (DRC) implemented lockdown measures on 18 March 2020 [2]. These measures were associated with significantly increased stress due to confinement in homes, loss of income, and discontinuation of services potentially leading to increased Intimate Partners’ Violence (IPV) [3]. IPV is defined as any act of violence perpetrated or suffered in the context of an intimate relationship. It refers to physical and sexual violence, including rape, emotional abuse, and other types, such as controlling behaviors by an intimate partner [4,5,6].

IPV can have long-lasting psychological and health effects for victims and their families [7,8] including post-traumatic stress disorder [9]. Studies showed that children born from mothers who were victims of IPV during pregnancy tend to develop internalizing problems, such as depression, and externalizing issues, such as violence and hyperactivity [10]. In the majority of IPV cases, victims are highly vulnerable and may not report it by fear of cultural and social norms in the African context [11,12]. Under-reporting of IPV incidents has been found to be a common problem [13,14].

Already before the COVID-19 outbreak, IPV was rife in the majority of countries [15], and particularly in SSA; a Demographic and Health Survey (DHS) analysis showed that 36% of women in urban SSA had experienced IPV at least once during their lives [16]. While there were significant regional discrepancies, IPV was shown to be correlated to gender dynamics and marital power inequities based on traditional gender norms. Several studies have reported an increase in IPV during humanitarian crises, and many countries including high-income countries reported increases in IPV since the COVID-19 pandemic [15,17,18,19]. Moreover, in high-income countries such as France and Belgium, due to the disruption of access to some specialized services and healthcare, a great demand for supporting victims of IPV was observed [15].

During confinement, sexual partners who live together spend more time at home without going out for working or social activities. This may lead to boredom and cause misunderstandings, quarrels and violence between sexual partners [20]. In addition, the majority of people in the DRC survive via day-to-day activities in the informal sector. Thus confinement was a period of significant economic crisis for couples and families [21]. Especially those working in informal economies or who were self-employed [22]. With schools closed, children and parents were confined for entire days in limited spaces.

In this study we sought to describe the magnitude of IPV against women and to identify determinants of IPV in the DRC during a period of lockdown COVID-19 control measures.

## 2. Materials and Methods

### 2.1. Study Setting and Design

The study was conducted as part of a series of studies to monitor the adherence to COVID-19 preventive measures and the impact of the COVID-19 pandemic in Low and Lower-Middle Income Countries (LICs/LMICs), organized by the International Citizen Project COVID-19 (ICPCovid). A first online survey was conducted in the DRC from 23 April, to 8 June 2020 [23] and a second from 24 August 2020 to 8 September 2020. Results showed only moderate adherence to COVID-19 preventive measures. Despite physical distancing often not being respected, face mask use was only 41.4% during the first survey and 69% during the second survey [24]. For the current analysis, only responses to questions asked about IPV during the second survey were included. We hypothesized that younger women, with lower income and a low level of education would be more at risk of IPV experience during the COVID-19 lockdown period.

### 2.2. Study Instrument and Participants’ Recruitment

A web-based online questionnaire was developed (see Supplementary Materials) by the ICPCovid consortium in English and translated to French and adapted for online use in the DRC [23]. The questionnaire included questions on demographic characteristics such as age, sex, educational level, occupation, living conditions, and questions related to the IPV during the confinement period and type of IPV experienced.

Participants were recruited using convenience and snowball sampling methods. Initially, the hyperlink to the online questionnaire was disseminated via internet platforms, such as Facebook, and by WhatsApp and emails. Upon clicking on the link, potential participants were informed about the study objectives and data confidentiality and could provide their e-consent. Thereafter, consenting respondents were able to fill out the online questionnaire and submit their responses. Potential participants in different districts were contacted electronically, and respondents were encouraged to further share the link within their networks. To increase participation, we used study assistants who used social media to motivate potential participants in their network to participate in the survey. They also went out to physically meet and assist potential study candidates who had limited access to the internet or had difficulties in filling out the form [15]. The study assistants were asked to approach the first 60 people they met per day in targeted streets randomly selected. Transportation and mobile internet fees were reimbursed to the study assistants, and they observed strict COVID-19 preventive measures (physical distancing and face masking) while in the field. When needed, the study assistants shared their internet access to enable participants to access the online questionnaire. Study participants did not receive any financial support or incentive.

### 2.3. Study Variables

Our main study outcome consisted of any form of violence reported by any female participant during the COVID-19 confinement period (March through June 2020), summarized as a binary variable (IPV vs. non-IPV experience). The following types of violence were explored: physical violence, verbal violence, sexual violence, and psychological violence. Other variables which were investigated included the following:

- Age: this is the respondent’s age at the last birthday; this variable was categorized into four modalities: 18–<30 years, 30–39 years, 40–49 years, and ≥50.
- Gender: this was a dummy variable categorized into 2 modalities: male and female.
- Marital status: this was the civil status of the respondent, a nominal variable categorized into 5 modalities: married, single, divorced, common-law, and widower. This variable was later dichotomized into two modalities, either the respondent lives alone or the respondent married/living together with a sexual partner.
- Level of education: this is the highest level of education attained by the respondent; this variable had three modalities, namely primary school, secondary/baccalaureate, and university level.
- Religion: this is the religious belief of the interviewee. This variable was categorized into 5 modalities: none, Protestant/Adventist, Catholic, Pentecostal, and other religions. This variable was re-categorized into three groups: Catholic, Pentecostal, and others.
- Belonging to the health sector: this was a dichotomous variable describing whether the respondent was a student/worker in the health sector (yes vs. no).

### 2.4. Data Management, Processing and Analysis

A dataset was created from all completed questionnaires in the general database. The records of participants including for women of reproductive age and who had at least one sexual partner were extracted from the comprehensive survey database. The dataset was exported to a Microsoft Excel 2016 spreadsheet for cleaning and coding, and subsequently transferred to R version 3.5.3 (R Foundation for Statistical Computing, Vienna, Austria) for analysis.

Descriptive statistics were conducted. Categorical variables were summarized, using frequencies and proportions. Continuous variables were summarized, using mean and standard deviation (SD) if normally distributed, or median and interquartile range (IQR) otherwise.

In bivariate analysis, we tested for associations between various variables and IPV experience. All variables with a likelihood ratio *p*-value < 0.25 in bivariate regression were included in the multivariable analysis. The selected variables from the bivariate analysis were subjected to a backward stepwise selection process and a final model was selected based on the least value for the Akaike information criterion (AIC) [25].

Multivariable analysis was conducted by using logistic regression to investigate determinants associated with IPV experience in women during the COVID-19 confinement. Thus, we employed logistic regression with Generalized Estimation Equations (GEE) to control for correlation among study participants in the same province. We adopted the exchangeability assumption for the correlation structure even though GEEs are robust to misspecifications of the correlation structure within each province; hence, the cluster effect was controlled for each province. We also estimated the variance inflation factors to check for multi-collinearity. This was negligible, since values were less than 10 as a rule of thumb. The level of significance used was 5% and all tests were two sided. The association between dependent and independent variables was determined by adjusted odds ratios (AOR), with 95% confidence intervals (95% CI) and *p*-value < 0.05, to determine the statistical significance level of these factors.

### 2.5. Ethical Considerations

The study protocol was submitted and approved by the DRC National Ethics Committee. To ensure confidentiality, data were collected online anonymously and were only available to study investigators, using password-protected files. All participants provided an e-consent before submitting their responses.

## 3. Results

A total of 4160 people participated in the survey. Ten provinces were excluded from the analysis because they had less than 350 respondents [15], thereby excluding 29 respondents. Thus, 4131 participants including 2830 (68.5%) women from seven provinces were considered for this study, from seven provinces: Haut Katanga, Kasaï-Central, Kasaï-Oriental, Kinshasa, Congo Central, Kwilu, and North Kivu. Among all the women, 2002 (70.7%) were living with at least one sexual partner (Table 1).

### 3.1. Sociodemographic and Health Characteristics of Women with Sexual Partners

In the sample of women living with sexual partner(s) (*n* = 2002), the mean age was 36.3 (SD: 8.2). Most of these women (65.8% of participants) were younger than 40 years. Almost half of the women were affiliated with the Catholic church; three out of four women had attained secondary level education (74.7%) and were living in urban areas (76.2%). One woman out of five was pregnant (19.4%) and 6.1% were healthcare workers (Table 2).

### 3.2. Level of Intimate Partners’ Violence

Out of 2002 women included in the analysis, 235 (11.7%) reported any form of IPV during the COVID-19 confinement period in the DRC.

In each province the percentage is calculated based on the number of women who reported any type of IPV. Other IPV included violence, such as psychological IPV.

### 3.3. Types of Intimate Partners’ Violence

**Figure 1:**
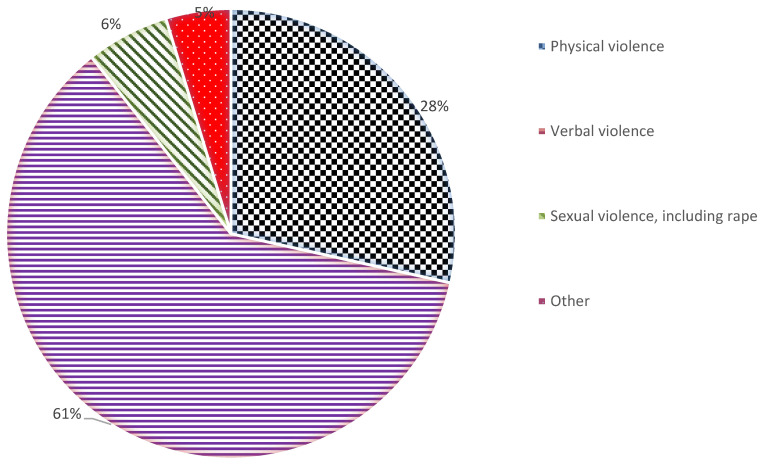
Type of Intimate Partners’ Violence experienced during the COVID-19 lockdown.

The most experienced type of IPV was verbal violence (Figure 1), reported by 143 (60.9%) women with highest percentages in Kwilu and Kasai-Oriental with 80.0% and 77.1%, respectively (Table 3). Physical violence was reported by 67 (28.5%) women with the highest percentage in Haut Katanga (56.1%), Kasai-Oriental (25.0%) and Kongo Central (25.0%). Sexual violence, including rape, was experienced by 14 women (6.0%) overall, with the highest percentage in Kongo-Central (8 women; 25.0%).

### 3.4. Factors Associated with Intimate Partners’ Violence

The multivariable logistic regression analysis with GEE estimation procedure assessing the factors associated with IPV during the COVID-19 lockdown revealed the following protective factors: being in the 30–39 and >50 years age groups (OR = 0.66, CI: 0.46–0.95; *p* = 0.026 and OR = 0.23, CI: 0.11–048; *p* < 0.001, respectively), living in urban setting (OR = 0.63, CI: 0.41–0.99; *p* = 0.047), and being in the middle socioeconomic class (OR = 0.48, CI: 0.29–0.79; *p* = 0.003) significantly decreased the odds for experiencing IPV (Table 4). However, a lower socioeconomic level (OR = 1.84, CI: 1.04–3.24; *p* = 0.035) and being pregnant (OR = 1.63, CI: 1.16–2.29; *p* = 0.005) or being uncertain of pregnancy status (OR = 2.01, CI: 1.17–3.44; *p* = 0.011) significantly increased the odds for experiencing IPV (Table 4).

## 4. Discussion

This study investigated IPV against women in seven provinces of DRC during the COVID-19 related lockdown. Of the 2002 women who participated in our online survey, 235 (12%) reported to have experienced any form of IPV since the start of the COVID-19 pandemic in the DRC.

The global pandemic of IPV existed before COVID-19, but several countries have reported an increase in cases of IPV, including serious cases that resulted in deaths [15]. In the context of SSA, data may be underestimated and under-reported due to cultural norms not considering some actions and abuses as violence [15].

We identified important disparities among provinces with regards to the level of IPV. The provinces of Congo-Central had the highest proportion of IPV (30.5%), followed by the provinces of Nord-Kivu and Kasai-Oriental, with 17.7% and 17.2%, respectively. The most prevalent type of violence was verbal abuse. The highest proportion of this type of IPV was observed in the provinces of Kwilu and Kasai-Oriental. The second most cited type of abuse was physical violence, with the highest proportion reported in Haut-Katanga province. The reason for this is not clear. The armed conflict in Grand Kasai has been cited as triggering violence, including gender violence [26]. Additional reasons may be gender inequalities mostly due to patriarchal lines as well as some norms and beliefs about masculinity and societal acceptance that conflicts can be solved by violence [15,27,28,29].

We speculate that gender inequities may be among the main factors in this situation [15,27,28,29]. Sexual violence, including rape, ranked third, with the highest proportion (57.1%) reported in the province of Kongo-Central.

The proportion of women who reported IPV in our study (11.7%) is lower than proportions reported in the literature prior to the COVID-19 pandemic. Indeed, in a study among 42,143 urban women, 15–49 years old, in 27 SSA countries who participated in Demographic and Health Surveys [16], the proportion of women who reported at least one form of IPV ranged from 10.8% in Comoros to 56.3% in DRC (highest level of all SSA participating countries). However, the recall period in our study was only about 4 months (the period of the lockdown), whereas the proportions reported in the DHS study related to lifetime periods. Some studies have reported an increase of the sexual violence among partners who used tobacco/smoke and/or drink alcohol [20]. In this study, we did not collect data on the alcohol consumption. However, we collected data about tobacco smoking which showed that Kinshasa and Kongo-Central provinces reported the highest proportion of participants who smoke, i.e., 96 (17%) and 76 (14%), respectively (Table 1).

Logistic regression analysis revealed several factors associated with the less IPV. Women aged 30 years and older tended to report less IPV. This is in line with other studies showing that women’s older age was associated with decreased IPV [30,31,32,33]. Several studies in SSA and elsewhere found that older women had better communication and negotiations skills with sexual partners concerning the use of family planning methods, such as condoms [30,31,32,33].

Women who lived in cities experienced less IPV. The urban mixing of cultures may induce a change of social norms [34]. Moreover, those who live in cities may have a higher level of education [4,15] and better access to prevention and support services. The management of IPV cases in cities may be improved by involving the local judicial system which would impose exemplary sanctions perpetrators of IPV, and this may have a positive impact on the reduction of IPV [15].

Women with a middle and high income level reported fewer events of IPV. Families living in poverty struggle to fulfill their existential needs, which can lead to conflict in couples [34,35]. In addition, participants from a middle socioeconomic level may have easier access to support facilities in case of IPV and are likely more exposed to awareness raising campaigns conducted by organizations and local authorities that are fighting against IPV [34,35].

Lower socioeconomic status and a lower level of education were associated with more IPV. Several studies showed that a low socioeconomic level was associated with low health outcomes, including IPV [34,35,36]. Poverty has been shown to be a main factor associated with IPV in SSA [37]. A decline of public protection and social services has affected many families living in urban SSA, and their ability to maintain a decent living [16]. This has the potential to create intra-family tensions triggering IPV [38].

Pregnant women and those who had doubts about their current pregnancy status had a high likelihood of experiencing violence. It is known that pregnancy causes physiological changes in women [39]. Meta-analysis synthesizing African studies on IPV against pregnant women yielded an overall prevalence of 15.23% (95% CI: 14.38 to 16.08%) [40]. Generally, the high prevalence of IPV during pregnancy in the African context is understood as a result of gender inequalities [39,41,42]. Because of confinement and due to limited access to family planning, the increased frequency of sexual intercourse may increase the number of unplanned pregnancies. The lockdown and its consequences, including insecurity and loss of income to afford pregnancy-related costs, may have led to conflicts and stress among couples [15,43,44,45].

Several limitations of our study must be acknowledged. First, self-reports may be influenced by recall bias and social desirability. Moreover, prevailing cultural norms could have led to under-reporting of the IPV. Under-reporting of IPV has been documented due to these methodological limitations [46]. Secondly, our study sample may not be representative of the national Congolese population in the provinces where the survey was done. This is a general problem of online surveys [47], since not everyone has an equal probability to participate due to significant differences in internet access. However, the link for the questionnaire was disseminated as broadly as possible in the 17 provinces of the DRC, using mixed channels approaches (social media platforms such as Facebook, and by using WhatsApp and emails), and study assistants facilitated the dissemination of the survey link. The survey was also advertised in public media covering the entire country to increase representativeness at country level. In the context of an emergency as the COVID-19 pandemic, the pragmatic approach of an online survey enabled us to quickly reach a relevant sample size in seven provinces of the DRC. More IPV was reported by persons of lower socioeconomic status and a lower education level. Therefore, because such persons were underrepresented in our sample, most likely the prevalence of IPV in the general population in the DRC will be higher. Given the cross-sectional design of our study and in the absence of a similar survey before the establishment of the lockdown measures, it is impossible to determine the causal relationship between these measures and the IPV. However, incidents of violence against women have increased worldwide since the lockdowns were implemented (UN Women, 2020) [48]. Nevertheless, the results of our study should only be considered as a starting point for further more in-depth research among a more representative sample of the Congolese society, using quantitative but also qualitative research methods.

## 5. Conclusions

Our study found a 11.7% level of IPV during the COVID-19 confinement period in the DRC. Taking into account the context and cultural norms in many provinces in the DRC, IPV cases could even be under-reported. The disparity between the provinces in terms of both IPV prevalence and different types depicts the complexity of the phenomenon. It is important that public-health decision-makers should be aware that strict lockdown measures may lead to increased poverty and increased IPV. Therefore, such measures should be implemented with great caution, taking into account the collateral damage they may cause. Mitigation measures to prevent a potential increase in IPV should be considered. Moreover, future research, including qualitative studies, is needed to identify the underlying multi-factorial reasons for IPV and to uncover the mechanisms leading to IPV. Such information is needed to develop and implement interventions to prevent IPV and support healthcare providers in reducing its harmful consequences, and transform victims into survivors. [49]

## Figures and Tables

**Table 1:** Participants’ characteristics by province.

Characteristics	Total (%)	Province							*p*-Value
		Haut-Katanga	Kasaï-Central	Kasaï-Oriental	Kinshasa	Kongo-Central	Kwilu	Nord-Kivu	
Total *n* (%)	4131 (100)	511 (12.4)	621 (15.0)	578 (14.0)	633 (15.3)	616 (14.9)	689 (16.7)	483 (11.7)	
Gender, *n* = 4134 (%)									
Male	1304 (31.6)	7 (1.4)	31 (5.0)	137 (23.7)	289 (45.7)	297 (48.2)	337 (48.9)	206 (42.6)	<0.001
Female	2827 (68.4)	504 (98.6)	590 (95.0)	441 (76.3)	344 (54.3)	319 (51.8)	352 (51.1)	277 (57.4)	
Women with partner, *n* = 2830 (%)									
Yes	2002 (73.9)	496 (98.4)	565 (95.8)	410 (93.0)	200 (58.1)	102 (32.0)	130 (36.9)	186 (67.2)	<0.001
No	738 (26.1)	8 (1.6)	25 (4.2)	31 (7.0)	144 (41.9)	217 (68.0)	222 (63.1)	91 (32.8)	

**Table 2:** Women’s sociodemographic characteristics (*n* = 2002).

Characteristics	Province							Total
	Haut-Katanga	Kasaï-Central	Kasaï-Oriental	Kinshasa	Kongo-Central	Kwilu	Nord-Kivu	
	*n* (%)	*n* (%)	*n* (%)	*n* (%)	*n* (%)	*n* (%)	*n* (%)	*n* (%)
**Total**	479 (23.9)	563 (28.1)	406 (20.3)	171 (8.5)	105 (5.2)	126 (6.3)	152 (7.6)	2002 (100.0)
**Age**								
<30	153 (31.9)	32 (5.7)	181 (44.6)	42 (24.6)	31 (29.5)	33 (26.2)	53 (34.9)	525 (26.2)
30–39	178 (37.2)	324 (57.5)	110 (27.1)	32 (18.7)	31 (29.5)	52 (41.3)	67 (44.1)	794 (39.7)
40–49	117 (24.4)	157 (27.9)	73 (18.0)	58 (33.9)	32 (30.5)	27 (21.4)	19 (12.5)	483 (24.1)
≥50	31 (6.5)	50 (8.9)	42 (10.3)	39 (22.8)	11 (10.5)	14 (11.1)	13 (8.6)	200 (10.0)
**Education**								
Primary	14 (2.9)	0 (0.0)	60 (14.8)	15 (8.8)	11 (10.5)	17 (13.5)	11 (7.2)	128 (6.4)
Secondary	433 (90.4)	485 (86.1)	298 (73.4)	115 (67.3)	37 (35.2)	67 (53.2)	61 (40.1)	1496 (74.7)
University	32 (6.7)	78 (13.9)	48 (11.8)	41 (24.0)	57 (54.3)	42 (33.3)	80 (52.6)	378 (18.9)
**Marital**								
Legally married/Cohabitation	479 (100.0)	563 (100.0)	406 (100.0)	163 (95.3)	100 (95.2)	116 (92.1)	151 (99.3)	1978 (98.8)
Single/Widow or Divorced	0 (0.0)	0 (0.0)	0 (0.0)	8 (4.7)	5 (4.8)	10 (7.9)	1 (0.7)	23 (1.2)
**Religion**								
Catholic	261 (54.5)	327 (58.1)	136 (33.5)	48 (28.1)	19 (18.1)	55 (43.7)	64 (42.1)	910 (45.5)
Protestant	193 (40.3)	20 (3.6)	200 (49.3)	42 (24.6)	26 (24.8)	22 (17.5)	49 (32.2)	552 (27.5)
Other	25 (5.2)	216 (38.4)	70 (17.2)	81 (47.4)	60 (57.1)	49 (38.9)	39 (25.7)	540 (27.0)
**Occupation**								
Jobless/Student	286 (59.7)	362 (64.3)	194 (47.8)	51 (29.8)	49 (46.7)	68 (54.0)	56 (36.8)	1066 (53.3)
With Job	193 (40.3)	201 (35.7)	212 (52.2)	120 (70.2)	56 (53.3)	58 (46.0)	96 (63.2)	936 (46.7)
**Healthcare work**								
No	455 (95.0)	560 (99.5)	384 (94.6)	157 (91,8)	72 (68.6)	107 (84.9)	145 (95.4)	1880 (93.9)
Yes	24 (5.0)	3 (0.5)	22 (5.4)	14 (8.2)	33 (31.4)	19 (15.1)	7 (4.6)	122 (6.1)
**Income category**								
Low	457 (95.4)	490 (87.0)	171 (42.1)	74 (43.3)	58 (55.2)	108 (85.7)	114 (75.0)	1472 (73.5)
Lower & middle	2 (0.4)	61 (10.8)	202 (49.8)	69 (40.4)	15 (14.3)	11 (8.7)	5 (3.3)	365 (18.2)
High/Upper middle	20 (4.2)	12 (2.1)	33 (8.1)	28 (16.4)	32 (30.5)	7 (5.6)	33 (21.7)	165 (8.2)
**Living area**								
Urban/town	213 (44.5)	563 (100.0)	387 (95.3)	132 (77.2)	87 (82.9)	2 (1.6)	142 (93.4)	1526 (76.2)
Suburban/rural	266 (55.5)	0 (0.0)	19 (4.7)	39 (22.8)	18 (17.1)	124 (98.4)	10 (6.6)	476 (23.8)
**Pregnant woman**								
Yes	355 (74.1)	478 (84.9)	296 (72.9)	113 (66.1)	77 (73.3)	96 (76.2)	102 (67.1)	1517 (75.8)
Non	98 (20.5)	85 (15.1)	97 (23.9)	39 (22.8)	21 (20.0)	13 (10.3)	37 (24.3)	390 (19.5)
Not sure	26 (5.4)	0 (0.0)	13 (3.2)	19 (11.1)	7 (6.7)	17 (13.5)	13 (8.6)	95 (4.8)
**Smokers**								
No	439 (91.6)	560 (99.5)	403 (99.3)	161 (94.2)	99 (94.3)	116 (92.1)	148 (97.4)	1926 (96.2)
Yes	40 (8.4)	3 (0.5)	3 (0.7)	10 (5.8)	6 (5.7)	10 (7.9)	4 (2.6)	76 (3.8)

**Table 3:** Type of Intimate Partners’ Violence reported by women, by province.

Characteristics	Province						
	Haut-Katanga *n* = 479	Kasaï-Central *n* = 563	Kasaï-Oriental *n* = 406	Kinshasa *n* = 171	Kongo-Central *n* = 105	Kwilu *n* = 126	Nord-Kivu *n* = 152
	***n* (%)**	***n* (%)**	***n* (%)**	***n* (%)**	***n* (%)**	***n* (%)**	***n* (%)**
Total IPV	41 (8.6)	16 (2.8)	70 (17.2)	29 (17.0)	32 (30.5)	20 (15.9)	27 (17.8)
Verbal IPV	13 (31.7)	12 (75.0)	54(77.1)	13 (44.8)	16 (50.0)	16 (80.0)	19 (70.4)
Physical IPV	23 (56.1)	4 (25.0)	16 (22.9)	7 (24.2)	8 (25.0)	4 (20.0)	5 (18.5)
Sexual IPV	2 (4.9)	0 (0.0)	0 (0.0)	4 (13.8)	8 (25.0)	0 (0.0)	0 (0.0)
Other IPV	3 (7.3)	0 (0.0)	0 (0.0)	5 (17.2)	0 (0.0)	0 (0.0)	3 (11.1)

**Table 4:** Factors associated with Intimate Partners’ Violence (*n* = 2002).

Variables	Modalities	OR Crude	IC_95%_ OR Crude	OR Adjusted	IC_95%_ OR Adjusted	*p*-Value
Age	Less than 30	1		1		
	30–39	0.59	0.42–0.84	0.66	0.46–0.95	0.0261
	40–49	0.67	0.46–0.98	0.75	0.50–1.11	0.1479
	50 and more	0.21	0.10–0.42	0.23	0.11–0.48	0.0001
Religion	Catholic	1		1		
	Protestant	1.50	1.07–2.12	1.31	0.92–1.88	0.1371
	Other	1.40	0.96–2.04	1.20	0.81–1.79	0.3695
Education level	Primary	1		1		
	Secondary	0.65	0.41–1.02	0.98	0.59–1.64	0.9496
	University	0.41	0.24–0.70	0.78	0.41–1.47	0.4402
Residential setting	Suburban/rural	1		1		
	Urban/Town	0.47	0.31–0.72	0.63	0.41–0.99	0.0479
Income category	High/Upper	1		1		
	Middle income	0.55	0.34–0.87	0.48	0.29–0.79	0.0038
	Low income	2.32	1.39–3.88	1.84	1.04–3.24	0.0357
Being pregnant	No	1		1		
	Yes	1.81	1.31–2.51	1.63	1.16–2.29	0.0053
	Do not know	2.47	1.49–4.09	2.01	1.17–3.44	0.0112
Smoking	No	1		1		
	Yes	2.52	1.43–4.45	1.75	0.94–3.29	0.0792

## Data Availability

Data are available upon reasonable request. Data are available on the International Consortium (International Citizen Project COVID-19 (ICPcovid): http://www.icpcovid.com accessed on 29 Jun 2020) website and could be used by other investigators upon request. De-identified participant data are available. My ORCID identifier is 0000-0002-3022-4879, and my email is john.ditekemena@unikin.ac.cd.

## Supplementary Materials

The following are available online at https://www.mdpi.com/article/10.3390/ijerph18105108/s1, RDC Covid-19 Questionnaire.
